# Oxytocin varies across the life course in a sex-specific way in a human subsistence population

**DOI:** 10.1073/pnas.2509977122

**Published:** 2025-12-15

**Authors:** Abigail E. Colby, Dominik C. Jud, Valerie Baettig, Jordan S. Martin, Camila Scaff, Michael D. Gurven, Benjamin C. Trumble, Bret A. Beheim, Paul L. Hooper, Daniel K. Cummings, Hillard Kaplan, Jonathan Stieglitz, Arnulfo Cary Ista, Adrian V. Jaeggi

**Affiliations:** ^a^Human Ecology Group, Institute of Evolutionary Medicine, University of Zürich, Zürich 8057, Switzerland; ^b^Institute of Evolutionary Anthropology, University of Zürich, Zürich 8057, Switzerland; ^c^Department of Fish Ecology and Evolution, Swiss Federal Institute of Aquatic Science and Technology, Kastanienbaum 6047, Switzerland; ^d^Laboratoire de Sciences Cognitives et Psycholinguistique, Départment d’études Cognitives, École Normale Supérieure, École des Hautes Études en Sciences Sociales, Centre National de la Recherche Scientifique, Université Paris Sciences & Lettres, Paris 75005, France; ^e^Department of Anthropology, University of California Santa Barbara, Santa Barbara, CA 93106; ^f^School of Human Evolution and Social Change, Center for Evolution and Medicine, Institute of Human Origins, Arizona State University, Tempe, AZ 85287; ^g^Department of Human Behavior, Ecology and Culture, Max Planck Institute for Evolutionary Anthropology, Leipzig 04103, Germany; ^h^Department of Anthropology, University of New Mexico, Albuquerque, NM 87131; ^i^Economic Science Institute, Argyros School of Business and Economics, Chapman University, Orange, CA 92866; ^j^Toulouse School of Economics, Toulouse 31080, France; ^k^Manguito, Beni Province, Manguito, Bolivia

**Keywords:** oxytocin, life history, aging, health, hormones

## Abstract

Oxytocin, a hormone linked to reproduction and health, may mediate life-history trade-offs across the human life course. Yet, how oxytocin naturally varies with age remains poorly understood. Here, working with the Tsimane, forager-horticulturalists of lowland Bolivia, we collected the largest sample of oxytocin measurements to date (n = 1,242 samples, n = 405 individuals, age = 2 to 84 y, 51% female), and i) examined how oxytocin varies throughout the life course in females and males, and ii) investigated potential drivers of age- and sex-specific variation. Our sample provides rare insight into the relationship between oxytocin and age in a context where energy is limited and trade-offs between reproduction and somatic maintenance are more salient. We found that oxytocin follows a nonlinear sex-specific trajectory throughout the life course. In females, oxytocin levels were high during the reproductive years and declined in the early to midforties, a pattern largely explained by breastfeeding and, to a lesser extent, childcare and good self-rated health. In males, oxytocin was low in early adulthood but high in old age, and although higher oxytocin was linked to good self-rated health, this did not explain the rise in oxytocin in later life. These findings suggest that oxytocin is instrumental for reproduction and caregiving in females and may also be associated with health in both males and females. Together, our work highlights oxytocin’s broader biological significance across the human life course, suggesting that it may play a pivotal role in coordinating age- and sex-specific trade-offs involving reproduction and health.

## Significance

The hormone oxytocin is linked to reproduction, social bonding, and health. To date, no single study has investigated oxytocin in both sexes across the life course. Working with the Tsimane of lowland Bolivia—a subsistence population with high fertility and high pathogen burden—we collected the largest ever dataset of oxytocin measurements. We show that females have higher oxytocin during their reproductive years, when they are breastfeeding and caring for children. Conversely, males showed low oxytocin during those years, but high oxytocin in older age. Our results suggest that oxytocin may mediate reproduction and caregiving in females and health in both sexes.

Hormone levels naturally fluctuate across the life course, and through coordinated effects on behavior and physiology, these fluctuations can shift energy investment toward reproduction at certain times and toward survival and repair during others (1). As such, hormones are thought to mediate life-history trade-offs, shaping how energy is allocated among growth, reproduction, and somatic maintenance in many organisms, including humans (2, 3). Oxytocin (OT), a neuropeptide hormone known for its role in birth and milk letdown (4), has gained recognition as a facilitator of social bonds (5, 6) and health (7). And, due to its positive impact on numerous physiological systems, OT has received attention as a potential therapeutic treatment for age-related conditions (7–9). Given its wide-ranging effects on health and reproduction, OT may play an adaptive role in reproduction and healthy aging, particularly in contexts with strong energetic constraints—such as high fertility, high infectious disease burden, and limited caloric intake—as was common for most of human evolutionary history (3).

Despite widespread interest in OT, little is known about how it varies throughout life, and by extension, what trade-offs it might mediate (10). To date, our best understanding of age-related changes in OT is a meta-analysis that combined differently aged samples from several published studies. In this study, OT was higher in samples from older individuals; however, subgroup analyses did not replicate this age pattern. Specifically, studies that extracted samples, as is typically recommended (11), showed a nonsignificant but negative relationship between age and OT and studies that measured OT from unextracted samples showed no meaningful relationship between age and OT. Moreover, most of the studies included in the analyses were conducted among low fertility populations and excluded samples from pregnant and postpartum individuals, despite the strong link between OT and reproduction in females (12). A more comprehensive understanding of age- and sex-specific variation in OT is needed, particularly among populations that have higher fertility and energetic constraints.

In females, OT is thought to facilitate breastfeeding and mother–offspring bonds (4, 13, 14). OT levels are higher in breastfeeding mothers than in bottle-feeding mothers (15), and during pregnancy and the postpartum period, they are associated with the quality of mother–offspring bonds and affectionate parenting behavior (16–18). Taken together, previous work linking OT to breastfeeding and caregiving in human mothers suggests that OT would be elevated during periods of life when such caregiving behaviors are more frequent.

In human males, OT may also mediate parenting (14). However, the association between parenting and OT in males is more complicated. In industrialized populations, fathers demonstrate higher OT levels than nonfathers (6), and OT is positively correlated with certain parenting behaviors (18), suggesting OT may mediate a shift from mating effort to parenting effort in humans. In the Tsimane, a forager-horticulturalist population in lowland Bolivia, hunting duration was positively correlated with OT levels upon returning home—a response that may help reinforce parenting behavior following a prolonged absence from the family (19). Among the Bodongo, a small-scale fishing-farming population in the northern Republic of Congo, men seen as good caregivers showed no difference in OT and lower testosterone compared to men seen as relatively poorer caregivers. However, men seen as good providers had relatively low OT, but high testosterone, compared to men seen as poorer providers (20). These findings suggest that OT’s role in fatherhood is context-dependent and may reflect population-specific trade-offs between provisioning and direct care that may affect testosterone and OT in men (5). Importantly, OT may also facilitate grandparental care, however, at present, little to no work has been reported on this topic. Given that OT has also been linked to food sharing (21) and social cognition (5), in contexts, such as the Tsimane, where older generations frequently provide food, childcare, and cultural knowledge (22–25), OT could modulate these behavioral investments, leading to higher OT in later life.

Beyond reproduction and caregiving, OT is linked to various health-related traits in both males and females (7). More specifically, OT is associated with immune system regulation (26, 27), wound healing (28), and cardiovascular function (29–31). In males, OT supports age-related muscle maintenance (9). In females, evidence from controlled laboratory studies in animal models, as well as observational studies and randomized controlled trials in humans, has shown that higher OT is linked to reduced menopause symptoms. Consequently, OT has been proposed as both a potential therapeutic target and biomarker of health during menopause (8). In subsistence populations like the Tsimane, who experience high pathogen burden (22) yet maintain notably good cardiovascular health compared to industrialized populations (32), OT may promote healthy aging.

Here, we take an exploratory approach using the largest sample to date of endogenous OT measurements in humans to i) describe how OT varies across the life course in females and males, and ii) investigate the potential drivers of age- and sex-specific variation in OT. Our cross-sectional sample (n = 1,242 samples, n = 405 individuals, age = 2 to 84 y, 51% female) (*SI Appendix*, Fig. S1, Table S11) was collected working with the Tsimane of lowland Bolivia—a population that experiences high fertility, prolonged breastfeeding, high pathogen burden, and lives in small communities with tightly knit social networks cooperating in daily subsistence and childcare (23, 33). As these conditions more closely resemble those experienced throughout human evolutionary history compared to industrialized populations, this approach enables greater insight into how natural selection might have shaped human biology and health (22). We measured urinary OT, which has the benefit of providing an integrated signal of OT released over several hours—thus better capturing the pulsatile release of OT during breastfeeding and other OT-related behaviors, compared to plasma. A disadvantage of urinary OT is that it may not reflect OT levels in the brain, as central and peripheral levels are not correlated during all contexts (34); as such, our data may better reflect OT’s peripheral, physiological functions, than its central, behavioral functions.

## Results

### Oxytocin Across the Life Course in Females and Males.

**Fig. 1.**
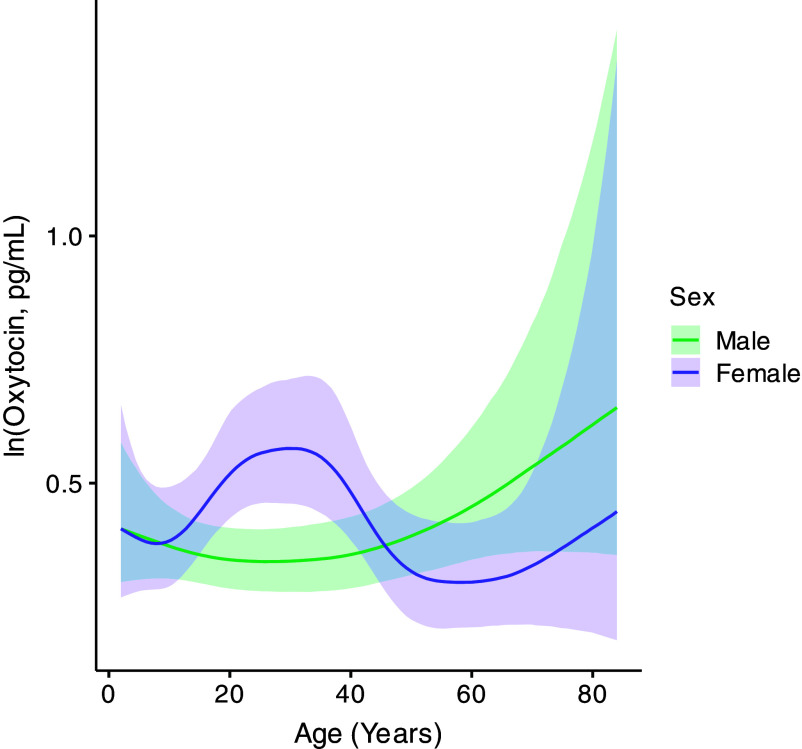
OT across the life course in males and females. The solid line indicates the posterior mean across age, and the shaded region represents the 95% CI of the posterior distribution.

In females, we found a strong nonlinear relationship between age and OT (spline standard deviation (SDS) = 2.36, 95% CI = 0.55 to 5.78; *SI Appendix*, Table S1). Specifically, females showed low OT levels in childhood, a rise in the early teenage years, and a peak during the late twenties to early thirties. OT then declined in the early to midforties and remained relatively low throughout the postreproductive lifespan, except for a slight increase from the midsixties onward (Fig. 1). In males, the relationship between age and OT was also nonlinear (SDS = 0.72, 95% CI = 0.05 to 2.05; *SI Appendix*, Table S1), with OT levels at their lowest in the twenties, followed by a gradual rise throughout the rest of adulthood, reaching their highest levels in old age (Fig. 1). Furthermore, in this model, we found moderate repeatability in OT levels: across repeated measures from the same individual, variation between individuals (participant ID SD = 0.61, 95% CI = 0.55 to 0.69; *SI Appendix*, Table S1) was roughly equal to variation within individuals (sigma = 0.63, 95% CI = 0.60 to 0.67; *SI Appendix*, Table S1). Additionally, females overall exhibited higher OT levels than males (sex = “Male” compared to “Female”: estimate = −0.20, 95% CI = −0.36 to −0.04; posterior probability (PP) female > male = 0.99; *SI Appendix*, Fig. S2*A* and Table S1).

### Drivers of Age- and Sex-Specific Variation Across the Life Course.

**Fig. 2.**
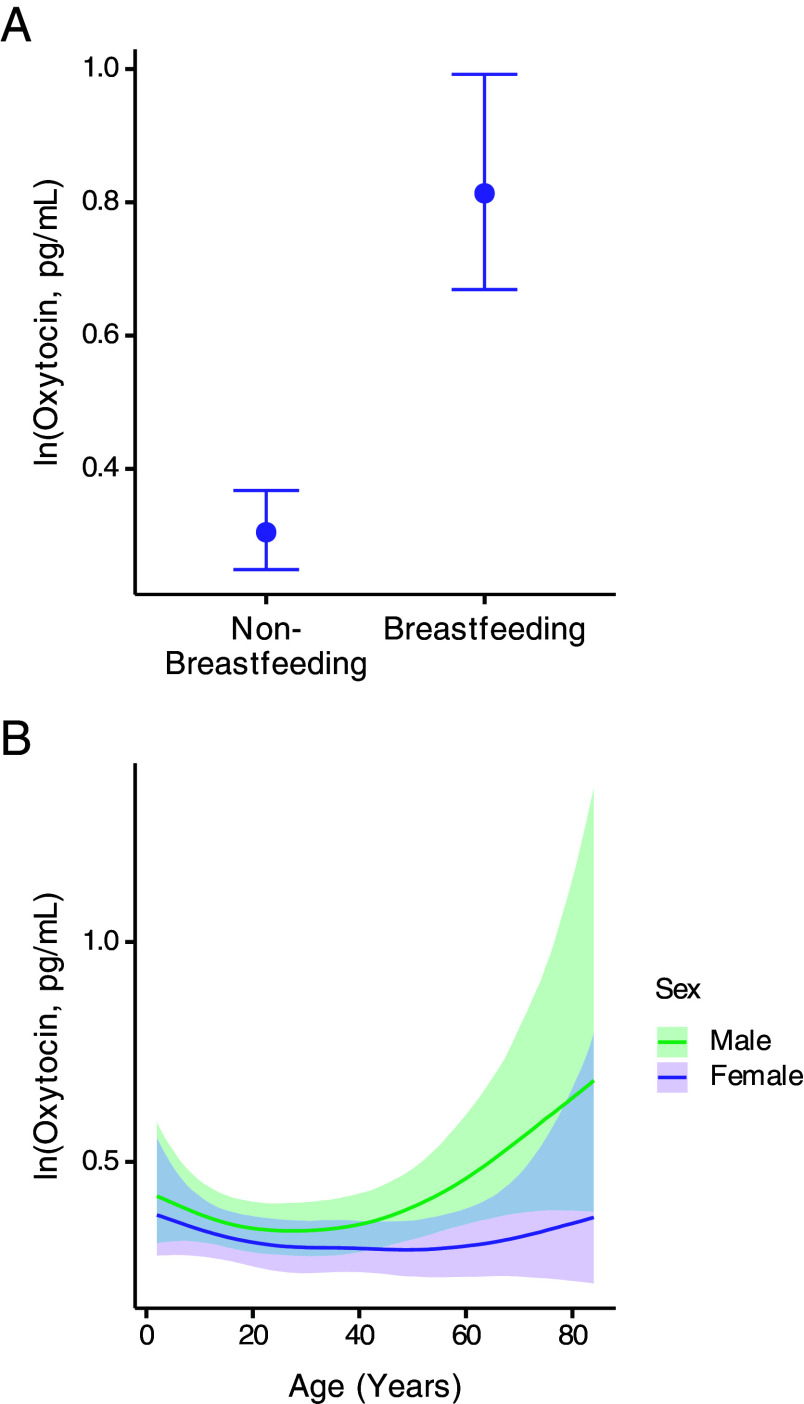
(*A*) OT levels by breastfeeding status in females. Points illustrate posterior means, and vertical lines indicate 95% CI with horizontal caps representing the upper and lower bounds. (*B*) OT levels by age and sex from the breastfeeding model. The solid line indicates the posterior mean across age, and the shaded region represents the 95% CI of the posterior distribution.

The age- and sex-specific patterns of OT raise the question of what functions OT might facilitate at the times when it peaks—specifically, in females during their reproductive years and in males during old age. For women, the well-established role of OT in breastfeeding and mother–infant bonding made it a prime candidate. To investigate the role of breastfeeding on OT levels, we included whether a participant was currently breastfeeding as a predictor in the model. Results showed that OT levels were higher among breastfeeding females than nonbreastfeeding females (breastfeeding = “Yes” compared to “No”: estimate = 0.98, 95% CI = 0.77 to 1.21; PP breastfeeding > nonbreastfeeding = 1.00; Fig. 2*A* and *SI Appendix*, Table S2). Moreover, inclusion of breastfeeding status as a model predictor flattened the rise and peak in OT during the female reproductive years (SDS = 0.61, 95% CI = 0.02 to 2.07; Fig. 2*B* and *SI Appendix*, Table S2), indicating that this peak was primarily mediated by breastfeeding. Additionally, inclusion of breastfeeding reversed the difference in OT levels between females and males (sex = “Male” compared to “Female”: estimate = 0.18, 95% CI = 0.02 to 0.34; PP female > male = 0.01; *SI Appendix*, Fig. S2*B*, Table S2), suggesting that sex differences in OT were also primarily mediated by breastfeeding.

**Fig. 3.**
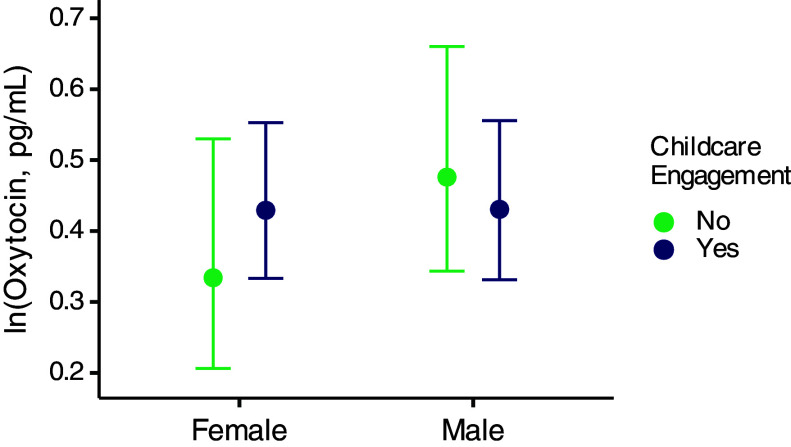
OT levels by childcare engagement. Points illustrate posterior means, and vertical lines indicate 95% CI with horizontal caps representing the upper and lower bounds.

While men cannot breastfeed, engaging in childcare is also known to involve OT and may mediate the peak in older males. To investigate the effect of childcare other than breastfeeding (e.g., playing, holding) on OT levels in males and females, we asked participants whether they had engaged in childcare during the few hours prior to the sample collection and included the self-reported answer associated with that urine sample into the breastfeeding model. In females, we found that having engaged in childcare was linked to higher OT (childcare = “Yes” compared to “No”: estimate = 0.26, 95% CI = −0.19 to 0.73; PP childcare > no childcare = 0.87; Fig. 3 and *SI Appendix*, Table S3), suggesting that OT may mediate childcare engagement in females regardless of breastfeeding status. In males, engagement in childcare was not linked to higher OT (childcare = “Yes” compared to “No”: estimate = −0.10, 95% CI = −0.40 to 0.21; PP childcare > no childcare = 0.26; Fig. 3 and *SI Appendix*, Table S3), suggesting that OT does not mediate childcare engagement or explain the age-related OT variation in males.

Even if individual bouts of childcare did not explain the peak in OT in older males, perhaps OT reflects more prolonged exposure to (grand) paternal duties. To investigate this, we coded individuals with at least one living (and typically coresiding) biological child as parents and individuals with at least one biological grandchild residing in the same community as grandparents. In males, we found no meaningful relationship between parent status and OT (father = “Yes” compared to “No”: estimate = −0.04, 95% CI = −0.42 to 0.36; PP father > nonfather = 0.41; *SI Appendix*, Fig. S3 and Table S4), suggesting little to no relationship between OT and fatherhood among the Tsimane. Similarly, in females, we found that when controlling for breastfeeding, mothers had OT levels indistinguishable from those of nonmothers (mother = “Yes” compared to “No”: estimate = −0.09, 95% CI = −0.46 to 0.29; PP mother > nonmother = 0.32; *SI Appendix*, Fig. S3 and Table S4). Additionally, in both males and females, individuals with at least one grandchild living in the same community showed slightly lower OT than individuals that did not have at least one grandchild living within their community (grandfather = “Yes” compared to “No”: estimate = −0.23, 95% CI = −0.62 to 0.14; PP grandfathers > nongrandfathers = 0.11; grandmother = “Yes” compared to “No”: estimate = −0.23, 95% CI = −0.59 to 0.11; PP grandmothers > nongrandmothers = 0.10; *SI Appendix*, Fig. S4 and Table S5), again suggesting that grandparental investment does not explain the rise in OT in older males. Moreover, we investigated how maternal vs. paternal grandparent status related to OT and found nearly identical results to the previous analysis that investigated grandparent status broadly (*SI Appendix*, Figs. S5 and S6 and Tables S6 and S7). However, very few individuals had paternal grandchildren but not maternal grandchildren living in the same community as the Tsimane are not primarily patrilocal.

**Fig. 4.**
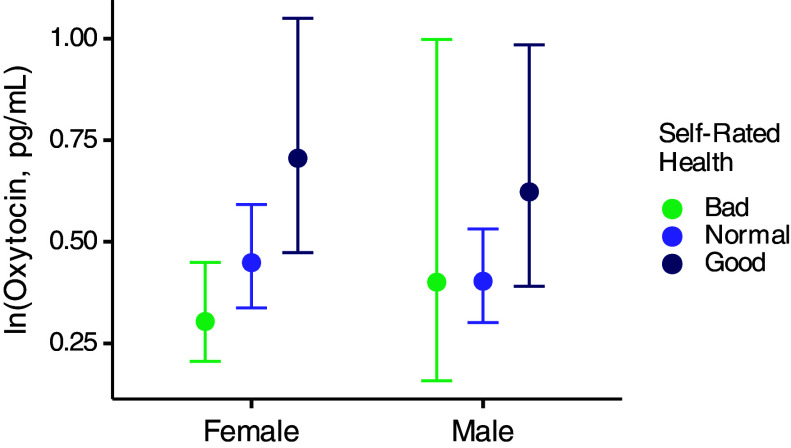
OT levels by self-rated health status. Points illustrate posterior means, and vertical lines indicate 95% CI with horizontal caps representing the upper and lower bounds.

Finally, to test the association of OT with health, we asked people to rate their health compared to others of the same age in their community. When adding self-rated health to the model, we found that females with good self-rated health had higher OT than females with normal or bad self-rated health (self-rated health = “Good” compared to “Normal”: estimate = 0.45, 95% CI = 0.05 to 0.85, PP good > normal = 0.99; self-rated health = “Good” compared to “Bad”: estimate = 0.84, 95% CI = 0.35 to 1.33, PP good > bad = 1.00; Fig. 4 and *SI Appendix*, Table S8). Similarly, males with good self-rated health had higher OT than males with normal or bad self-rated health (self-rated health = “Good” compared to “Normal”: estimate = 0.44, 95% CI = −0.04 to 0.92, PP good > normal = 0.96; self-rated health = “Good” compared to “Bad”: estimate = 0.45, 95% CI = −0.54 to 1.45, PP good > bad = 0.81; Fig. 4 and *SI Appendix*, Table S8). However, this association with health also did not fully explain the rise of OT in older males (and females).

## Discussion

Our study demonstrates that peripheral OT levels have a distinct, sex-specific trajectory throughout the life course in a subsistence population, peaking at about age thirty in females and in old age for males. Moreover, OT levels are linked to reproductive status in females and self-reported health status in both males and females, suggesting that OT may mediate trade-offs involving reproduction and maintenance throughout life.

### Oxytocin Across the Life Course in Females and Males.

In females, elevated OT during the twenties and thirties is consistent with previous work highlighting its role as a reproductive and parenting hormone, as this pattern of OT mirrors the reproductive life history of Tsimane women and the typical age trajectory of other female reproductive hormones (35). During these years, the Tsimane birth on average nine children (36), breastfeed each child for an average of 19.2 mo (37) during an average of 2.2 y between births, and engage in high amounts of direct care toward young children compared to other decades of life (38). Tsimane women typically have their first child by age twenty and have their last in the late thirties or early forties. Interestingly, our findings regarding the age trajectory of OT in females do not align with previous work that did not find elevated OT among samples from reproductive aged females (12). However, this may be because this analysis excluded studies that included pregnant and postpartum women and that most studies included were derived from industrialized populations with lower fertility and shorter breastfeeding durations.

In males, OT was low in the twenties and highest in old age. It has been suggested that OT-related events in life, such as becoming a parent and grandparent, may leave a lasting effect on the OT system, resulting in elevated levels in older age (12). However, theoretical arguments (39) and empirical evidence (40) tend to emphasize the impact of early life experiences on the OT system, rather than that of later-life events, such as becoming a grandparent. Regardless, we were unable to find a direct predictor of the OT peak in older males in the way that breastfeeding explained the peak in reproductive-aged females. Furthermore, we are unable to test whether higher OT observed in older males is due to a rise in later life, or if higher OT males live longer than lower OT males, potentially due to OT’s effects on health.

### Drivers of Age- and Sex-Specific Variation Across the Life Course.

Our investigation into the drivers of OT suggests that breastfeeding and caring for children are primary drivers of OT variation throughout the life course in females. Specifically, we found that breastfeeding was linked to higher OT, which mediated the pronounced peak in OT levels in the reproductive years. These findings are consistent with OT’s known role in lactation (4) and other studies that reported higher OT among breastfeeding compared to bottle-feeding mothers (15). Despite OT’s known role in lactation, some studies found no difference between breastfeeding and nonbreastfeeding mothers (16), but this is likely due to the pulsatile nature of OT release during breastfeeding, which may be missed in the plasma or saliva samples used in these studies (as OT has a short half-life in these mediums), but not in the urine samples used here (as OT is steadily cleared into urine and our participants urinated only a few times per day, but breastfed frequently) (34).

In females, having engaged in childcare was also linked to higher OT regardless of the individual’s breastfeeding status, suggesting that not only breastfeeding but caring for children more broadly may be mediated by OT, even when measured in the periphery. This lends support to previous work that links affectionate parenting behavior (16) and quality of mother–offspring bonds (16, 17) to OT in mothers regardless of breastfeeding status. Interestingly, in males, we found no strong link between having engaged in childcare and OT. Moreover, we found no meaningful difference in OT between fathers and nonfathers, suggesting that among the Tsimane OT may not mediate a shift in effort from mating to parenting as is reported in industrialized populations (6, 18). Interestingly, our findings are also inconsistent with the interpretation of previous work among the Tsimane which suggested that higher OT after longer hunting trips prepares men to engage in direct childcare (19), though in subsistence populations food provisioning is an important component of paternal investment. Indeed, our findings are consistent with those from the Bodongo, which found that fathers seen as good providers have low OT compared to fathers seen as poorer providers, but no difference between fathers seen as good or poor direct caregivers (20). Moreover, the contrast between our findings and those of Mascaro et al. (6) emphasizes the importance of context when investigating OT and traits that are complex and may be culturally specific, such as fatherhood. For example, among the Tsimane, exposure to and care for children is common for a childless person compared to a childless person in most Western contexts. For Tsimane men, status is driven by traits such as physical formidability, influence over others in the community, and the ability to provide food for their family, rather than by willingness to engage in direct childcare (41–43). Moreover, the Tsimane are known to practice sexual divisions of labor (41) and, compared to women, men in their twenties and thirties engage in very little direct childcare (38). Taken together, this suggests that father status does not explain the age pattern in male OT levels we found in this study, and that OT may not mediate parenting effort among Tsimane men as it is thought to among men in a Western context. Moreover, patterns of OT levels across the lifespan may not be universal, but rather influenced by societal norms. Thus, future studies that investigate how OT varies across the life course—ideally longitudinally—in other societies, industrialized and not, could further illuminate the role of OT in paternal investment.

In later life, social roles and behaviors shift among the Tsimane. For example, Tsimane men in their sixties spend more time in their community than younger men (38). Moreover, older men have enhanced skill mastery (24) and know more stories (25) such that they are often regarded as cultural experts and teachers among their families and communities. These changes in behavior in later life could be partially facilitated by shifts in OT, as OT mediates social cognition and learning (5, 44); these behavioral factors could perhaps explain the upward trajectory of OT in older-aged males, though this was not directly tested. Previous work has indicated that OT and testosterone may be inversely correlated (5, 20) and, among some populations, testosterone decreases gradually with age after forty (45, 46). Therefore, if OT and testosterone are inversely correlated, a decline in testosterone with age could explain the higher OT among older males in our sample. However, previous work has indicated that testosterone does not decline with age among the Tsimane as is observed in other, mostly industrialized, populations (47). Moreover, in previous work, OT and testosterone were positively correlated among Tsimane men (19), which suggests that this is not the most likely explanation for higher OT observed among older males. However, because we do not have testosterone measures for these specific samples, we cannot say definitively that this is not a contributing factor toward higher OT observed in older men.

Moreover, higher OT could be associated with greater social connectedness, as has been shown in industrialized populations (48, 49). We did not test this explanation directly; however, given that social connectedness is linked to better mental health and reduced mortality risk (50–52), and that subjective well-being declines with age in the Tsimane (53), this possibility warrants further investigation. This interpretation aligns with our findings on self-rated health’s association to OT, as an individual’s social environment is argued to modulate the impact of OT on health (7).

Interestingly, grandfathers—males with at least one biological grandchild living in the same community—had lower OT than nongrandfathers, when controlling for age, and grandfather status did not explain age-related changes in OT among males. Rather than OT rising in response to grandparental duties, these findings may instead reflect variation in life-history strategy mediated, in part, by OT. More specifically, if men with higher OT levels delay reproduction and become fathers at a later age, they will likely also become grandfathers at a later age, resulting in grandfathers having lower OT than age-matched nongrandfathers. This pattern could emerge from trade-offs between growth and somatic maintenance, and reproduction (54). If higher OT promotes health and longevity, as is suggested by the association between OT and self-rated health in this study, it may also be linked to later age at first reproduction due to greater investment in growth and development in early life. Consistent with this explanation, some previous work has linked worse health to earlier sexual debut and earlier age at first reproduction in both men and women (55). However, evidence for life-history trade-offs is notoriously mixed (56), including in humans in general (54), and the Tsimane in particular (57, 58), so this explanation remains speculative until further investigation.

We found that good self-rated health was linked to higher OT among the Tsimane in males and females. These findings are consistent with previous work linking OT to various health traits (7) and suggests that OT may mediate and promote somatic maintenance throughout life, especially in old age. We used self-rated health because, despite being a subjective measure, it is a powerful predictor of health, morbidity, mortality, and clinical outcomes (59, 60). In the Tsimane, aging is marked by increased physiological dysregulation (61) and inflammation (62). However, inflammaging (i.e., age-associated increase in chronic inflammation) is notably different among the Tsimane than in industrialized populations (63). Despite experiencing high levels of inflammation due to high pathogen burden, the Tsimane have the lowest prevalence of coronary atherosclerosis of any studied population (32), as well as lower levels of dementia (64) and markedly slower age-related brain volume decrease compared to populations in Europe and the United States (65). Given the relationship between self-rated health and OT, the higher OT levels observed in older age in males (and females) may reflect a shift in OT secretion that could promote somatic maintenance in response to the increased physiological dysregulation and inflammation experienced in later life. Nonetheless, incorporating self-rated health within the model did not explain the higher levels of OT in older males, suggesting that more nuanced health-related measures (e.g., specific biomarkers of inflammation) are needed to fully explain this pattern.

Our findings suggest that in old age, females have comparatively lower OT than males. This pattern indicates that males and females may have different strategies for promoting longevity, shaped by distinct reproductive requirements. For example, parous women show less brain aging than nulliparous women, suggesting that pregnancy and the postpartum period have lasting positive effects on brain health (66). Moreover, breastfeeding has prolonged effects on metabolism (67) and inflammation (68), providing protection against chronic diseases such as type 2 diabetes (69, 70). Additionally, previous work indicates that breastfeeding is associated with reduced incidence of cardiovascular and Alzheimer’s disease (71, 72), suggesting that extended breastfeeding—potentially through the beneficial effects of more sustained OT throughout the reproductive years—may confer long-term health benefits without the need to maintain high OT in later life. This possibility is especially relevant given global declines in fertility and shortened breastfeeding durations, highlighting potential unforeseen consequences of these shifts on women’s long-term health.

A few caveats arise from the fact that our study was cross-sectional rather than longitudinal. Namely, we are unable to say whether OT levels increased with older age or if the older participants in our study had relatively high OT throughout their life. In the latter case, observed higher OT levels in old age could reflect a survivor bias in the sample—specifically, individuals with higher OT may live to an older age than those with lower OT and make up most older aged individuals in the study. This explanation may be particularly relevant for males but could also explain the small rise in OT in older females. Presently, we are unable to perform a survival analysis (73) to investigate this question due to the cross-sectional nature of our data. However, the average remaining life expectancy for Tsimane men aged 25 to thirty—when OT is at its lowest—is approximately 43 years (74), extending well into the ages when OT is markedly higher. In other words, most men who reach adulthood are expected to experience both the low and high OT ages observed in our sample, suggesting that survival bias is unlikely the sole explanation for our finding that older males have comparably high OT. Similarly, we cannot infer with confidence that OT causes better health. It is equally possible that the observed relationship between self-rated health and OT reflects overall differences in genetic quality or access to resources, allowing some individuals to express both better health and higher OT levels, or that individuals with higher OT are simply subjectively experiencing or reporting their health as better. Thus, while our finding that good self-rated health is linked to higher OT in a high fertility, high pathogen burden, subsistence population suggests that OT may mediate somatic maintenance to promote health and longevity, further studies, ideally longitudinal, are needed to support this.

Overall, our results highlight the importance of considering sex-specific hormonal strategies over the life course and suggest that OT may be a key mediator of evolutionary trade-offs involving reproduction and health.

## Materials and Methods

Our study was approved by the Ethics Committee of the University of Zurich (#23.03.13) and the Institutional Review Board at University of California Santa Barbara (#3-21-0652) and approved by the Tsimane Government (Grand Consejo) and the communities visited during fieldwork. Prior to participation, informed verbal consent was obtained from participants after the study was explained to them in their native language through a bilingual research assistant.

### Data Collection.

Working with the Tsimane of lowland Bolivia, we collected urine samples (n = 1,242 samples, n = 405 individuals, age = 2 to 84 y, 51% female) (*SI Appendix*, Fig. S1, Table S11) and associated interviews that asked for breastfeeding status, parent status, engagement in childcare, and self-rated health status, during two field seasons in March–April 2023 and September–December 2023. Moreover, we collected demographic interviews (used to estimate age, breastfeeding status, parent status, and grandparent status) for most adult participants between November 2023 and November 2024. To estimate participant age, we used self-reports from demographic interviews, community census records, and the demographic database maintained by the Tsimane Health and Life History Project (THLHP) (see Gurven et al. (74) for more details on age estimation). We were unable to acquire a date of birth for one participant that contributed one sample to our dataset. In this case, age was estimated visually by one of the authors (A.V.J.).

To ascertain breastfeeding status and parent status, we used a conservative, multisource approach. Specifically, we drew from demographic interviews, self-reports, and behavioral observations, as well as the demographic database maintained by the THLHP (for more detail, see *SI Appendix*, Tables S12, S14). We assessed childcare engagement with an interview question asked directly after sample collection: “Have you cared for children in the last several hours (or since last urination)?” In this case, what constituted childcare was determined by the participant and was most often interpreted as direct care (e.g., playing, feeding, holding) (*SI Appendix*, Table S13). We determined grandparent status from demographic interviews. Because our interest was in those who could provide resources or direct care to their grandchildren consistently, only participants with at least one biological grandchild living in the same community were coded as “Yes” for grandparent status (*SI Appendix*, Tables S15-S17). To assess self-rated health, we asked participants to rate their own health on a three-point scale (Bad, Normal, Good) compared to others of the same age in their community (*SI Appendix*, Table S18). First, participants were asked about their present health status (i.e., currently ill or injured) and how their current health compared to their health in the previous year. Then, participants were asked to identify a few age-matched individuals in their community. Finally, we asked the participants to rate their health in comparison to that of their peers. Despite being a subjective measure, self-rated health is a reliable predictor of health, clinical outcomes, morbidity, and mortality (59, 60).

### Measuring Oxytocin.

Within 15 to 120 min (typically under 60 min) of being collected from the participant, the urine samples were frozen in a liquid nitrogen tank and kept frozen—with dry ice (during transport from Bolivia to the United States) or in a -80 freezer—until they were thawed for laboratory analyses conducted between June 2023 and November 2024. OT was measured using radioimmunoassay at the Wisconsin Primate Research Center (WNPRC) (75) at the University of Wisconsin-Madison in Madison, WI, USA. Prior to assay, solid-phase extraction was performed. The immunoassay used in this study was the Phoenix Pharmaceuticals (Burlingame, CA) Oxytocin Radioimmunoassay kit (catalog # RK-05-01). For a complete description of the radioimmunoassay, see the methods section of Gerred & Kapoor (75).

To account for variation in urine concentration across samples, OT values were corrected for specific gravity (SG), measured using a refractometer, and with the following formula: SG-corrected OT concentration = raw OT concentration x (SG_population mean_ − 1.0/SG_sample_ − 1.0) (76). In cases where the OT value was estimated below or above the detection limits of the radioimmunoassay and could not be extrapolated using the standard curve of the assay, the samples were censored in statistical models (beneath the detection limit = left-censored, above the detection limit = right-censored) with the specific gravity corrected detection limit for each sample as the value for OT.

### Statistical Analyses.

All analyses were performed in R (version 4.5.1) (77).

We investigated age- and sex-related differences in OT throughout the life course using generalized linear multilevel models (GLMMs) and the brms package in R (78). We included mean-centered age with a sex interaction, sex, fieldwork, and context (first morning void or not first morning void—“morning” or “daytime”) as fixed effects and participant ID as a random effect to account for repeated measures across participants as most contributed more than one sample. Specifically, for age, we used a spline function with a sex interaction to investigate nonlinear relationships between age and OT in both sexes separately. In this model and subsequent models used to investigate the drivers of OT variation, we used a log normal distribution and specified weakly regularizing priors (intercept: normal (mean = 0, SD = 2); regression coefficients: normal(mean = 0, SD = 1); random effects: exponential (2)) that allow for parameter estimate flexibility and consideration of outliers in the data (79, 80). We ran the model for 4,000 iterations across 4 chains, including a 1,000-iteration warm-up period for each chain. To ensure accurate sampling and avoid divergent transitions, we set the adapt_delta to 0.9999 and increased the max_treedepth to 15.

To investigate potential drivers of age- and sex-specific variation in OT, we ran additional models that included breastfeeding status, engagement in childcare, parent status, grandparent status, and/or self-rated health status as predictor variables. Bayesian regression models estimate a posterior distribution for each parameter. Here, we mainly report the mean and 95% credible intervals (CI) of these posteriors (*SI Appendix*, Tables S1–S10), but note that the 95% CI threshold holds no particular meaning in Bayesian inference and should not be used to dichotomize results as significant or not (80). Instead, our confidence in a particular result is best expressed as the proportion of the posterior distribution that supports a result, e.g. the proportion > 0 for a positive association; we therefore report these posterior probabilities (or “PP”) for all relevant associations, and these should be interpreted probabilistically. For example, the entire posterior distribution for the slope of the breastfeeding predictor was > 0, indicating that we can be 100% certain that breastfeeding females have higher OT levels, given the data and model assumptions.

## Supplementary Material

Appendix 01 (PDF)

## Data Availability

The R code used for the analyses in this study can be found on Github: https://github.com/abby-colby/oxytocin-age-variation (81). Data availability is restricted for ethical reasons. The Human Ecology Group of the University of Zurich adheres to the CARE Principles for Indigenous Data Governance (https://www.gida-global.org/care) (82). These principles intend that Indigenous communities i) have sovereignty over how data are shared; ii) are the primary gatekeepers determining ethical use; iii) are actively engaged in the data generation; and iv) derive benefit from data generated and shared use. The Human Ecology Group is also committed to the FAIR Principles for scientific data management (https://www.go-fair.org/fair-principles/) (83) and will therefore help facilitate data sharing requests that adhere to the CARE principles. Requests for data reuse can be sent to Prof. Adrian Jaeggi (adrian.jaeggi@iem.uzh.ch) or Dr. Camila Scaff (camila.scaff@iem.uzh.ch) and should take the form of an application that minimally details the exact uses of the data and the research questions to be addressed, procedures that will be employed for data security and individual privacy, potential benefits to the study communities and procedures for assessing and minimizing stigmatizing interpretations of the research results. Requests for data reuse will require institutional ethics approval and will be reviewed by an Advisory Council composed of Tsimane community members and the Human Ecology Group leadership.
