# Supplementary material for: Oxytocin varies across the life course in a sex-specific way in a human subsistence population
Source: Proc Natl Acad Sci U S A. 2025 Dec 15;122(51):e2509977122. doi: 10.1073/pnas.2509977122 (PMC12745800; doi:10.1073/pnas.2509977122)
Supplement: Supplementary file 1 — Appendix 01 (PDF) [file pnas.2509977122.sapp.pdf]

PNAS

Supporting Information for

Oxytocin varies across the life course in a sex-specific way in a human subsistence population

Abigail E. Colby[a,1], Dominik C. Jud[a,b], Valerie Baettig[a], Jordan S. Martin[a,c], Camila Scaff[ad], Michael D. Gurven[e], Benjamin C. Trumble[f], Bret Beheim[g], Paul L. Hooper[h], Daniel K. Cummings[i], Hillard Kaplan[i], Jonathan Stieglitz[j], Arnulfo Cary Ista[k] & Adrian V. Jaeggi[a]

[a]Institute of Evolutionary Medicine, University of Zürich, Zürich, 8057, Switzerland
[b]Institute of Evolutionary Anthropology, University of Zürich, Zürich, 8057, Switzerland
[c]Department of Fish Ecology and Evolution, EAWAG, Kastanienbaum, 6047, Switzerland
[d]Laboratoire de Sciences Cognitives et de Psycholinguistique (ENS, EHESS, CNRS, DEC), PSL University, Paris, 75005, France
[e]Department of Anthropology, University of California Santa Barbara, Santa Barbara, CA 93106, United States
[f]School of Human Evolution and Social Change, Center for Evolution and Medicine, Institute of Human Origins, Arizona State University, Tempe, AZ 85287, United States
[g]Department of Human Behavior, Ecology and Culture, Max Planck Institute for Evolutionary Anthropology, Leipzig, 04103, Germany
[h]Department of Anthropology, University of New Mexico, Albuquerque, NM 87131, United States
[i]Economic Science Institute, Argyros School of Business and Economics, Chapman University, Orange, CA 92866, United States
[j]Toulouse School of Economics, Toulouse, 31080, France
[k]Manguito, Beni Province, Bolivia

[1]**Corresponding Author**: Abigail E. Colby

Email: abigail.colby@iem.uzh.ch

Author Contributions: A.E.C. and A.V.J. designed research; A.E.C., A.V.J., D.C.J., V.B., J.S.M., C.S., M.D.G., B.C.T., B.B., P.L.H., D.K.C., H.K., J.S., A.C.I., performed research; A.E.C. and D.C.J. analyzed data; A.E.C. and A.V.J. wrote the paper.

Competing Interest Statement: The authors declare no competing interests.

Classification: Biological Science / Anthropology

Keywords: oxytocin, hormones, life history, aging, health

This PDF file includes:

Supporting Materials and Methods
Significance Statement in Spanish
SI Reference
Figures S1 to S6
Tables S1 to S18

Materials and Methods

Measuring Oxytocin

During the September-December 2023 field season (fieldwork = FW2), we collected at least 3mL of urine and acidified the samples with phosphoric acid (following the protocol of Gerred & Kapoor (1)). In March-April 2023 (fieldwork = FW1), we collected at least 1.5mL of urine and did not acidify the samples, thus, the radioimmunoassay was performed on 1.5mL of non-acidified urine, not 3mL of acidified urine – otherwise, the protocol for measuring oxytocin (OT) follows that described in Gerred & Kapoor (1). In all analyses, fieldwork was included as a fixed effect to control for field season related differences. The coefficients of variance (CV) for our radioimmunoassay were within an acceptable range of under 10% and 15%, for intra-assay and inter-assay variability, respectively across lots (FW1: Intra-Assay CV% = 7.06, Inter-Assay CV% = 8.78; FW2: Intra-Assay CV% = 4.899, Inter-Assay CV% = 7.087), suggesting high reproducibility using this method.

Breastfeeding Status

In our dataset, male participants, female participants under 14 (pre-menarche) and over 48 (post-reproductive) as well as female participants who had never given birth at the time of sample collection were classified as non-breastfeeding ("No").

To determine breastfeeding status in reproductive-age female participants (14-48 years (2)), we used a conservative, multi-source approach. Specifically, we used the date of each participant's last birth, acquired from demographic interviews and the Tsimane Health and Life History Project demographic database, together with participant self-reports collected during the March-April 2023 and September-December 2023 field seasons. Previous work indicates that, among the Tsimane, breastfeeding durations range from 5 to 42 months post birth (3). With this information, we used the following logic to classify breastfeeding status.

<u>Breastfeeding was coded as "Yes"</u>:

- the participant self-reported "Yes" for breastfeeding and the participant had given birth to a living child in the 42 months prior to sample collection,
- the participant did not give a self-report but had given birth to a living child in the 5 months prior to sample collection

<u>Breastfeeding was coded as "No"</u>:

- the participant self-reported "No" for breastfeeding and the participant had not given birth to a living child in the 42 months prior to sample collection,
- the participant did not give a self-report but had not given birth to a living child in the 42 months prior to sample collection

<u>Breastfeeding was coded as "NA"</u>:

- the participant self-reported "Yes" for breastfeeding but had not given birth to a living child in the 42 months prior to sample collection (i.e., breastfeeding duration exceeded previously reported maximum durations),
- the participant did not give a self-report but had given birth to a living child 5 to 42 months prior to sample collection (i.e., above the minimum and within the previously reported breastfeeding range, but no self-report to verify that they in fact were breastfeeding at the time of sample collection)

In some missing breastfeeding status cases, we could corroborate breastfeeding status with behavioral observations (i.e., time allocation records) and direct personal knowledge of the participant. When breastfeeding status was uncertain ("NA"), the samples were removed from analyses investigating the association between breastfeeding on OT. Overall, our approach prioritized accuracy and minimized false positives for breastfeeding classification.

Parent Status

In our study, female participants were considered parents if they had given birth to a child and male participants were considered parents if they were the self-reported biological father of a child. If a participant was an adoptive parent, but not a biological parent, they were removed from analyses that investigated parent status (n=4 samples, n= 2 individuals). Moreover, if a participant did have biological children, but explicitly stated they did not live with any of those children, they were also removed from the model investigating the effect of parent status on OT (n=2 samples, n=1 individual).

Statistical Analyses

In addition to the model that used a smooth term (spline) to investigate age- and sex-related variation in OT (model 2), we ran two additional models to investigate the relationship between age and OT – specifically, a null model (model 0) that did not include our predictor variable of interest (i.e., age with a sex interaction) and a model with a linear age term with a sex interaction (model 1).

- **Model 0:** ot_sg_corrected | cens(censored) ~ 1 + sex + context + fieldwork + (1 | pid)
- **Model 1:** ot_sg_corrected | cens(censored) ~ 1 + age_centered*sex + context + fieldwork + (1 | pid)
- **Model 2:** ot_sg_corrected | cens(censored) ~ 1 + s(age_centered, by = sex) + sex + context + fieldwork + (1 | pid)

In all models, we used the structure and parameters described in the methods of the main text. We used LOO (leave-one-out) cross-validation to determine the best fit model. According to the LOO comparison, model 2, the model that included the spline function for age – to model nonlinear relationships – performed best (model 2: difference expected log predictive density (ΔELPD) = 0, standard error (SE) = 0) of all the models considered (model 1: ΔELPD = -3.7, SE = 2.9; model 0: ΔELPD = -2.0, SE = 2.7). However, using LOO, we encountered divergent transitions and high Pareto k diagnostic values, and confirmed the findings – that the model with a spline function for age (model 2) had the greatest predictive accuracy – using K-fold cross-validation (model 2: ΔELPD = 0, SE = 0; model 1: ΔELPD = -3.5, SE = 7.1; model 0: ΔELPD = -1.3, SE = 7.4) (4, 5).

Drivers of age- and sex-specific variation across the life course. All models included a smooth term for age by sex (s(age_centered, by = sex)) and a random intercept for participant ID ((1|pid)). Our fixed effects varied based on the predictor of interest for each model.

- **Breastfeeding Model:** ot_sg_corrected | cens(censored) ~ 1 + s(age_centered, by = sex) + sex + breastfeeding + context + fieldwork + (1 | pid)
- **Childcare Model:** ot_sg_corrected | cens(censored) ~ 1 + s(age_centered, by = sex) + sex*childcare + breastfeeding + fieldwork + (1 | pid)
  Note: *context is not included as a fixed effect because the childcare question was asked only after daytime sample collection.*
- **Parent Model:** ot_sg_corrected | cens(censored) ~ 1 + s(age_centered, by = sex) + sex*parent + breastfeeding + context + fieldwork + (1 | pid)
- **Grandparent Model:** ot_sg_corrected | cens(censored) ~ 1 + s(age_centered, by = sex) + sex*grandparent + breastfeeding + context + fieldwork + (1 | pid)
  Note: *maternal and paternal grandparent models use this model structure, but with grandparent replaced by maternal_grandparent or paternal_grandparent.*
- **Self-Rated Health Model:** ot_sg_corrected | cens(censored) ~ 1 + s(age_centered, by = sex) + sex*self-rated health + context + (1 | pid)
  Note: *fieldwork was not included as a fixed effect because the self-rated health question was only asked during fieldwork 2.*

Importancia del Estudio

La oxitocina es una hormona relacionada con la reproducción, los vínculos sociales y la salud. Hasta la fecha, ningún estudio ha investigado la oxitocina en ambos sexos a lo largo de toda la vida. En colaboración con los Tsimane' del Sur de Beni, un grupo indígena que vive de la subsistencia, con una alta tasa de fertilidad y un elevado nivel de exposición a patógenos, recopilamos el mayor conjunto de datos jamás registrado de mediciones de oxitocina. Mostramos que las mujeres tienen niveles más altos de oxitocina en sus años reproductivos, durante la lactancia y el cuidado de los hijos. Por el contrario, los hombres mostraron niveles bajos de oxitocina durante esos años, pero niveles altos en la vejez. Los resultados sugieren que la oxitocina puede mediar la reproducción y el cuidado de los hijos en las mujeres, así como la salud en ambos sexos.

References

1. K. Gerred, A fit-for-purpose validation of a commercial radioimmunoassay for measurement of human peripheral oxytocin. *Biochem. Biophys. Rep.* (2024).
2. "Life History" in *Human Behavioral Ecology*, 1st Ed., (Cambridge University Press, 2024), pp. 20–47.
3. A. Veile, M. Martin, L. McAllister, M. Gurven, Modernization is associated with intensive breastfeeding patterns in the Bolivian Amazon. *Soc. Sci. Med.* **100**, 148–158 (2014).
4. P.-C. Bürkner, **brms**: An *R* Package for Bayesian Multilevel Models Using *Stan*. *J. Stat. Softw.* **80** (2017).
5. A. Vehtari, A. Gelman, J. Gabry, Practical Bayesian model evaluation using leave-one-out cross-validation and WAIC. *Stat. Comput.* **27**, 1413–1432 (2017).

Figures

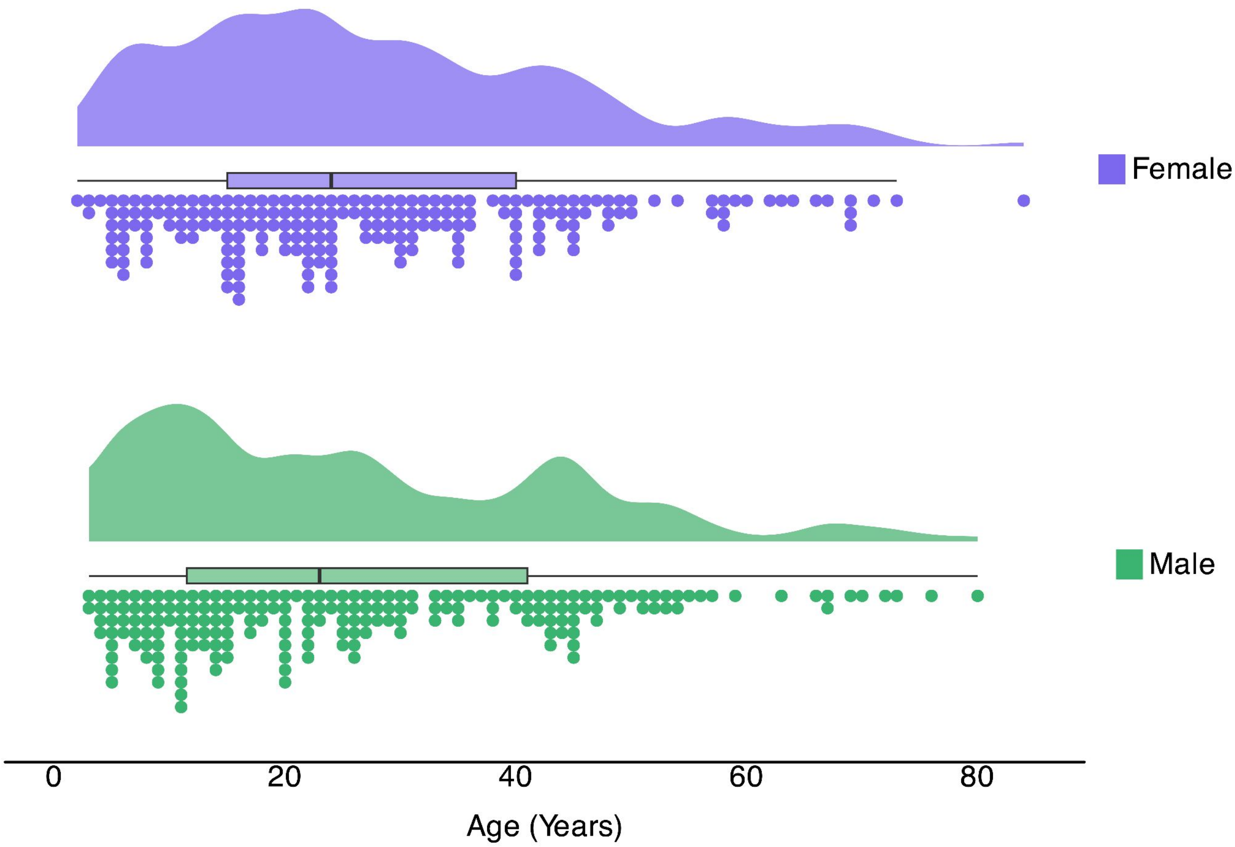

Fig. S1. Raincloud plot showing participant age distributions by sex. Because our sample includes repeated measures over 8 months, some participants provided samples at two ages (e.g., 30 and 31). In such cases, only the older age is displayed.

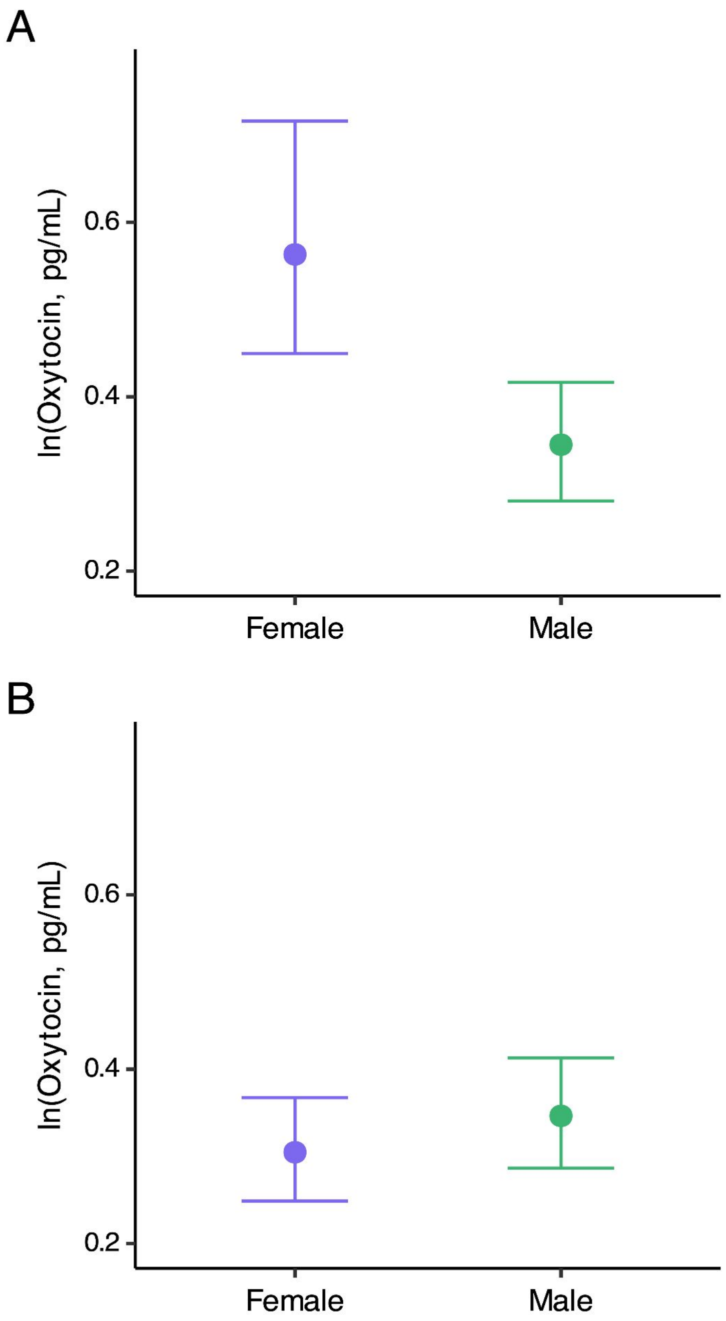

Fig. S2. (A) OT levels by sex from the age model and **(B)** OT levels by sex from the breastfeeding model. Points illustrate posterior means, and vertical lines indicate 95% CI with horizontal caps representing the upper and lower bounds.

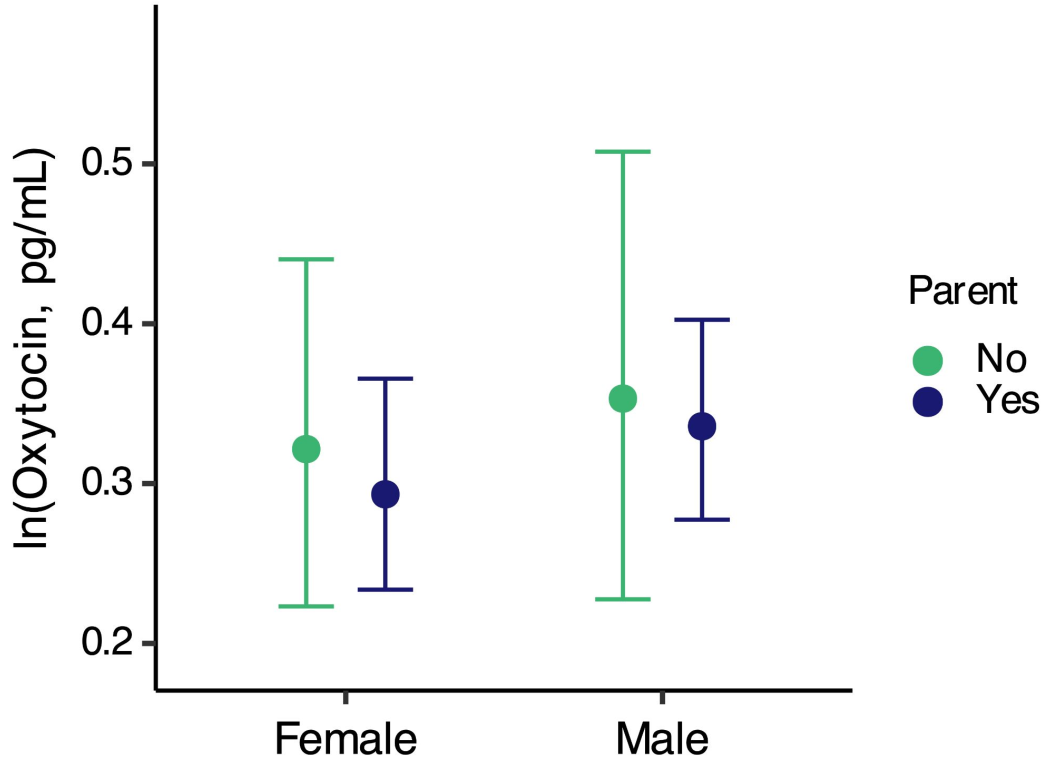

Fig. S3. OT levels by parent status. Points illustrate posterior means, and vertical lines indicate 95% CI with horizontal caps representing the upper and lower bounds.

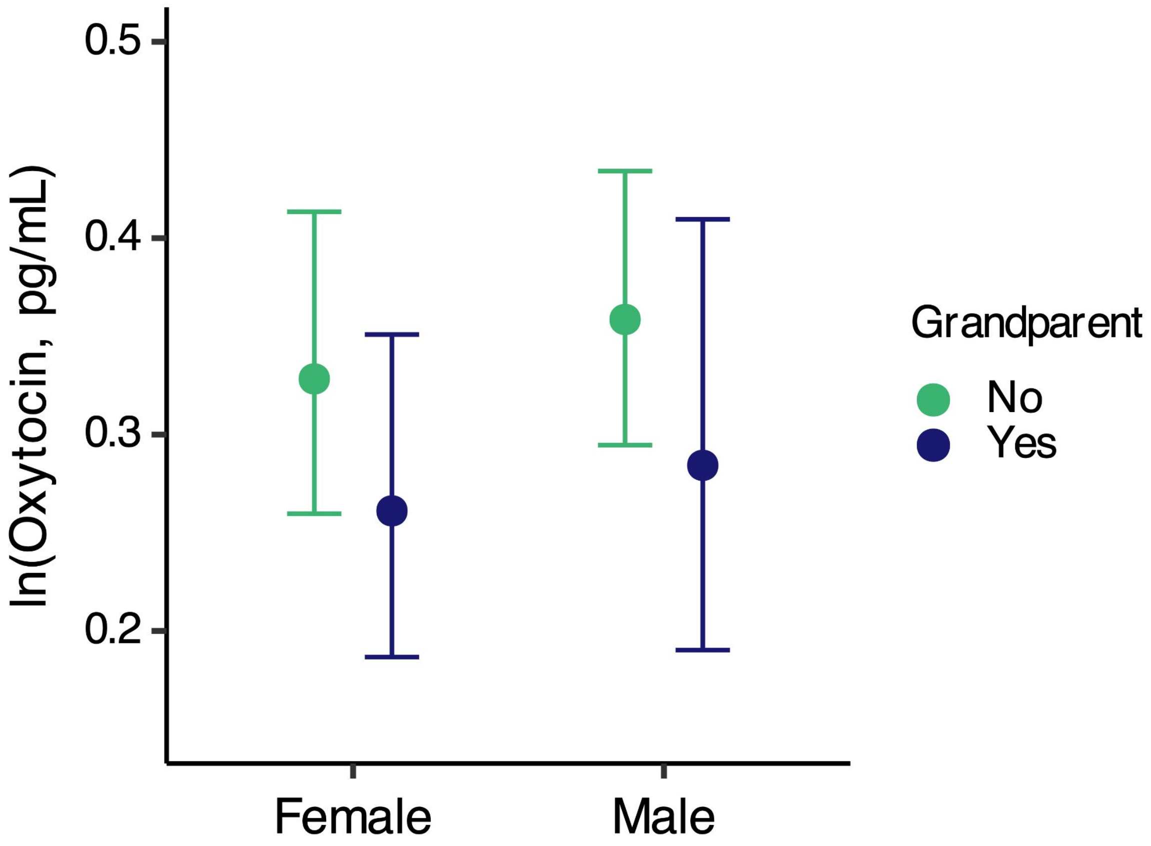

Fig. S4. OT levels by grandparent status. Points illustrate posterior means, and vertical lines indicate 95% CI with horizontal caps representing the upper and lower bounds.

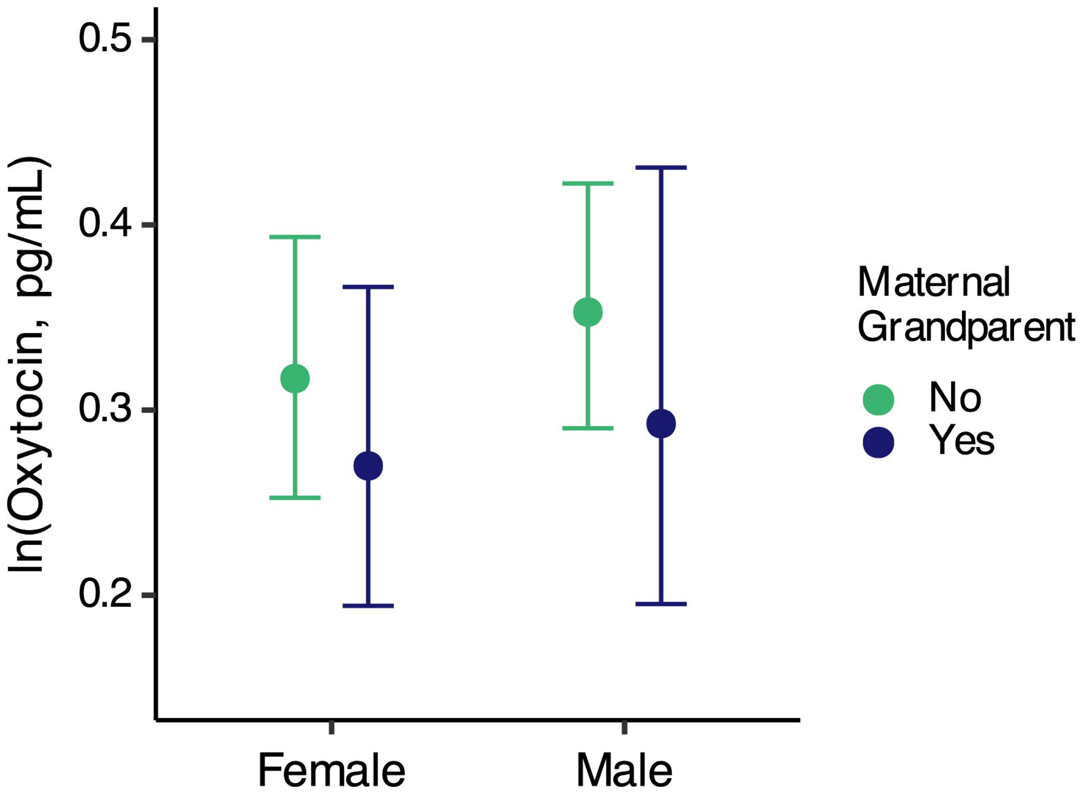

Fig. S5. OT levels by maternal grandparent status. Points illustrate posterior means, and vertical lines indicate 95% CI with horizontal caps representing the upper and lower bounds.

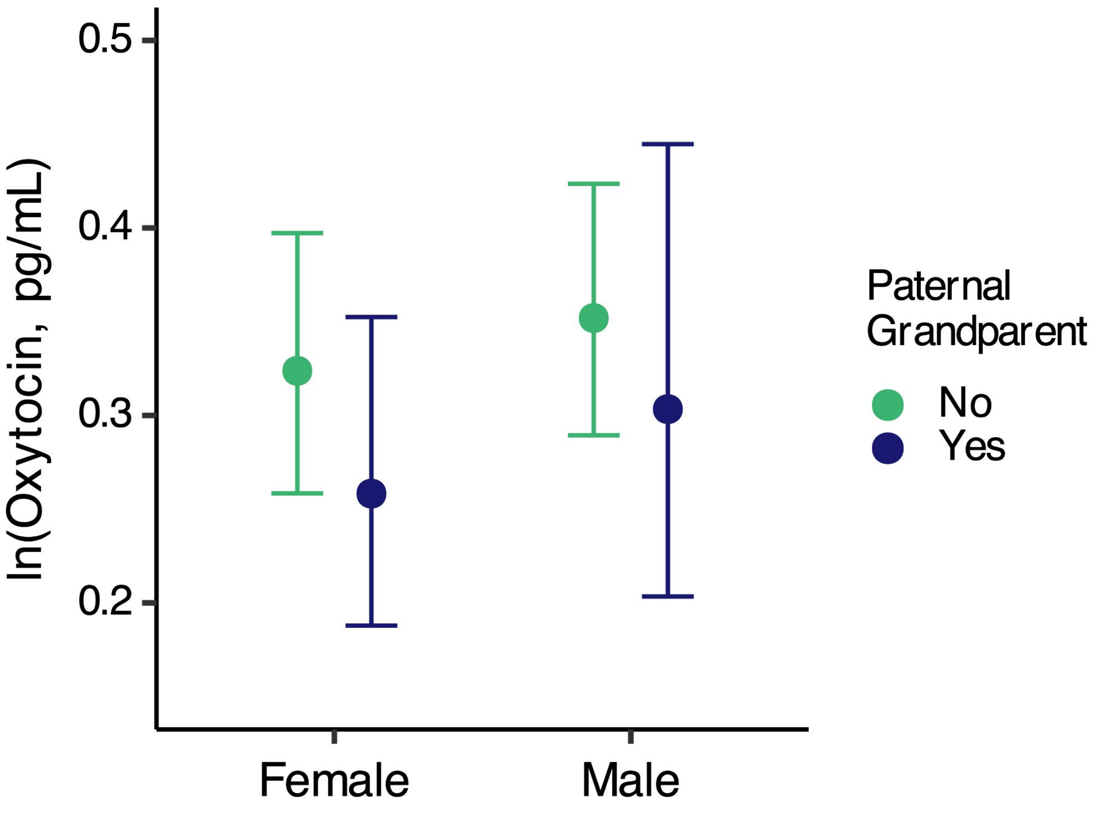

Fig. S6. OT levels by paternal grandparent status. Points illustrate posterior means, and vertical lines indicate 95% CI with horizontal caps representing the upper and lower bounds.

Model Summary Tables

Table S1. Age Model. The intercept refers to Sex = Female, Context = Morning, and Fieldwork = FW1.

Parameter	Estimate (β)	Estimate Error	Lower 95% CI	Upper 95% CI
Intercept	-1.00	0.07	-1.13	-0.86
Sex = Male	-0.20	0.08	-0.36	-0.04
Context = Daytime	0.14	0.04	0.05	0.22
Fieldwork = FW2	0.08	0.06	-0.04	0.20
Age*Sex = Female	0.08	0.96	-1.80	1.94
Age*Sex = Male	0.16	0.79	-1.47	1.68
Age*Sex = Female SDS	2.36	1.34	0.55	5.78
Age*Sex = Male SDS	0.72	0.53	0.05	2.05
Participant ID SD	0.61	0.04	0.55	0.69
Sigma (residual)	0.63	0.02	0.60	0.67

Table S2. Breastfeeding Model. The intercept refers to Sex = Female, Context = Morning, Fieldwork = FW1, and Breastfeeding = No. Highlighted parameters were added in this model.

Parameter	Estimate (β)	Estimate Error	Lower 95% CI	Upper 95% CI
Intercept	-1.36	0.08	-1.51	-1.21
Sex = Male	0.18	0.08	0.02	0.34
Context = Daytime	0.13	0.04	0.05	0.21
Fieldwork = FW2	0.08	0.06	-0.03	0.19
Age*Sex = Female	-0.13	0.73	-1.60	1.40
Age*Sex = Male	0.16	0.81	-1.50	1.72
Age*Sex = Female SDS	0.61	0.55	0.02	2.07
Age*Sex = Male SDS	0.74	0.50	0.09	2.01
Participant ID SD	0.51	0.04	0.44	0.58
Sigma (residual)	0.63	0.02	0.60	0.66
Breastfeeding = Yes	0.98	0.11	0.77	1.21

Table S3. Childcare Model. The intercept refers to Sex = Female, Fieldwork = FW1, Breastfeeding = No and Childcare = No. Highlighted parameters were added in this model.

Parameter	Estimate (*β*)	Estimate Error	Lower 95% CI	Upper 95% CI
Intercept	-1.31	0.23	-1.76=8	-0.86
Sex = Male	0.39	0.26	-0.11	0.90
Fieldwork = FW2	-0.11	0.10	-0.30	0.08
Age*Sex = Female	0.17	0.72	-1.31	1.56
Age*Sex = Male	0.49	0.87	-1.38	2.01
Age*Sex = Female SDS	0.48	0.49	0.02	1.80
Age*Sex = Male SDS	0.71	0.67	0.02	2.47
Participant ID SD	0.35	0.13	0.04	0.56
Sigma (residual)	0.66	0.06	0.55	0.78
Breastfeeding = Yes	0.99	0.15	0.70	1.29
Childcare = Yes	0.26	0.23	-0.19	0.73
Sex = Male*Childcare = Yes	-0.36	0.27	-0.90	0.18

Table S4. Parent Model. The intercept refers to Sex = Female, Context = Morning, Fieldwork = FW1, Breastfeeding = No, and Parent = No. Highlighted parameters were added in this model.

Parameter	Estimate (*β*)	Estimate Error	Lower 95% CI	Upper 95% CI
Intercept	-1.31	0.14	-1.61	-1.04
Sex = Male	0.14	0.21	-0.26	0.55
Context = Daytime	0.13	0.04	0.05	0.21
Fieldwork = FW2	0.08	0.06	-0.03	0.20
Age*Sex = Female	-0.01	0.73	-1.52	1.41
Age*Sex = Male	0.21	0.83	-1.54	1.74
Age*Sex = Female SDS	0.59	0.56	0.02	2.08
Age*Sex = Male SDS	0.76	0.53	0.07	2.09
Participant ID SD	0.52	0.04	0.45	0.59
Sigma (residual)	0.63	0.02	0.59	0.66
Breastfeeding = Yes	1.03	0.13	0.76	1.29
Parent = Yes	-0.09	0.19	-0.46	0.29
Sex = Male*Parent = Yes	0.04	0.27	-0.48	0.58

Table S5. Grandparent Model. The intercept refers to Sex = Female, Context = Morning, Fieldwork = FW1, Breastfeeding = No, and Grandparent = No. Highlighted parameters were added in this model.

Parameter	Estimate (β)	Estimate Error	Lower 95% CI	Upper 95% CI
Intercept	-1.27	0.10	-1.47	-1.07
Sex = Male	0.15	0.11	-0.08	0.38
Context = Daytime	0.12	0.04	0.05	0.21
Fieldwork = FW2	0.08	0.06	-0.04	0.19
Age*Sex = Female	0.02	0.77	-1.58	1.51
Age*Sex = Male	0.18	0.84	-1.54	1.77
Age*Sex = Female SDS	0.73	0.63	0.03	2.42
Age*Sex = Male SDS	0.87	0.54	0.14	2.21
Participant ID SD	0.51	0.04	0.44	0.59
Sigma (residual)	0.63	0.02	0.60	0.66
Breastfeeding = Yes	0.96	0.12	0.74	1.19
Grandparent = Yes	-0.23	0.18	-0.59	0.11
Sex = Male*Grandparent = Yes	-0.00	0.26	-0.51	0.50

Table S6. Maternal Grandparent Model. The intercept refers to Sex = Female, Context = Morning, Fieldwork = FW1, Breastfeeding = No, and Maternal Grandparent = No. Highlighted parameters were added in this model.

Parameter	Estimate (β)	Estimate Error	Lower 95% CI	Upper 95% CI
Intercept	-1.31	0.09	-1.50	-1.13
Sex = Male	0.17	0.10	-0.04	0.37
Context = Daytime	0.12	0.04	0.04	0.21
Fieldwork = FW2	0.08	0.06	-0.03	0.20
Age*Sex = Female	-0.03	0.76	-1.55	1.45
Age*Sex = Male	0.17	0.83	-1.52	1.76
Age*Sex = Female SDS	0.68	0.60	0.03	2.26
Age*Sex = Male SDS	0.84	0.55	0.13	2.27
Participant ID SD	0.52	0.04	0.45	0.59
Sigma (residual)	0.63	0.02	0.60	0.66
Breastfeeding = Yes	0.98	0.11	0.75	1.20
Maternal Grandparent = Yes	-0.16	0.17	-0.49	0.16
Sex = Male*Maternal Grandparent = Yes	-0.03	0.25	-0.52	0.47

Table S7. Paternal Grandparent Model. The intercept refers to Sex = Female, Context = Morning, Fieldwork = FW1, Breastfeeding = No, and Paternal Grandparent = No. Highlighted parameters were added in this model.

**Parameter**	**Estimate (β)**	**Estimate Error**	**Lower 95% CI**	**Upper 95% CI**
Intercept	-1.29	0.09	-1.48	-1.11
Sex = Male	0.14	0.10	-0.06	0.34
Context = Daytime	0.13	0.04	0.05	0.21
Fieldwork = FW2	0.08	0.06	-0.04	0.20
Age*Sex = Female	0.01	0.73	-1.47	1.44
Age*Sex = Male	0.16	0.82	-1.51	1.76
Age*Sex = Female SDS	0.63	0.58	0.03	2.10
Age*Sex = Male SDS	0.81	0.54	0.09	2.19
Participant ID SD	0.52	0.04	0.45	0.59
Sigma (residual)	0.63	0.02	0.60	0.66
Breastfeeding = Yes	0.94	0.12	0.71	1.18
Paternal Grandparent = Yes	-0.22	0.17	-0.55	0.11
Sex = Male*Paternal Grandparent = Yes	0.07	0.25	-0.43	0.56

Table S8. Self-Rated Health Model. The intercept refers to Sex = Female, Context = Morning, and Self-Rated Health = Normal. Highlighted parameters were added in this model.

**Parameter**	**Estimate (β)**	**Estimate Error**	**Lower 95% CI**	**Upper 95% CI**
Intercept	-0.91	0.11	-1.13	-0.69
Sex = Male	-0.15	0.16	-0.46	0.16
Context = Daytime	0.04	0.06	-0.07	0.15
Age*Sex = Female	0.05	0.96	-1.80	1.98
Age*Sex = Male	0.44	0.85	-1.35	1.98
Age*Sex = Female SDS	1.36	0.90	0.18	3.58
Age*Sex = Male SDS	0.69	0.65	0.02	2.47
Participant ID SD	0.61	0.06	0.50	0.72
Sigma (residual)	0.59	0.02	0.55	0.64
Self-Rated Health = Good	0.45	0.20	0.05	0.85
Self-Rated Health = Bad	-0.39	0.21	-0.79	0.01
Sex = Male*Self-Rated Health = Good	-0.01	0.31	-0.62	0.58
Sex = Male*Self-Rated Health = Bad	0.38	0.49	-0.57	1.35

Table S9. Age Model (Linear). The intercept refers to Sex = Female, Context = Morning, and Fieldwork = FW1.

Parameter	Estimate (β)	Estimate Error	Lower 95% CI	Upper 95% CI
Intercept	-1.01	0.07	-1.14	-0.87
Sex = Male	-0.17	0.08	-0.33	-0.01
Context = Daytime	0.14	0.04	0.06	0.22
Fieldwork = FW2	0.09	0.06	-0.03	0.21
Age	-0.00	0.00	-0.01	0.00
Age*Sex = Male	0.01	0.00	-0.00	0.02
Participant ID SD	0.64	0.04	0.57	0.71
Sigma (residual)	0.63	0.02	0.60	0.67

Table S10. Null Age Model. The intercept refers to Sex = Female, Context = Morning, and Fieldwork = FW1.

Parameter	Estimate (β)	Estimate Error	Lower 95% CI	Upper 95% CI
Intercept	-0.99	0.07	-1.13	-0.86
Sex = Male	-0.20	0.08	-0.35	-0.04
Context = Daytime	0.14	0.04	0.05	0.21
Fieldwork = FW2	0.09	0.06	-0.03	0.21
Participant ID SD	0.64	0.04	0.57	0.71
Sigma (residual)	0.63	0.02	0.60	0.67

Descriptive Information Tables

Table S11. Number of individuals and urine samples by sex included in the age model.

	***n* individuals**	***n* samples**
**female**	206	758
**male**	199	484
**total**	405	1242

Table S12. Number of individuals and urine samples by breastfeeding status included in the breastfeeding model.

	***n* individuals**	***n* samples**
**breastfeeding females**	58	168
**non-breastfeeding females**	136	558
**males**	199	484
**total**	392	1210

Table S13. Number of individuals and urine samples by childcare included in the childcare model.

	**female**		**male**		**total**	
	n individuals	*n* samples	*n* individuals	*n* samples	*n* individuals	*n* samples
**childcare**	113	153	63	79	176	232
**no childcare**	10	13	31	37	41	50
**total**	120	166	79	116	199	282

Table S14. Number of individuals and urine samples by parent status included in the parent model.

	**female**		**male**		**total**	
	n individuals	*n* samples	*n* individuals	*n* samples	*n* individuals	*n* samples
**parent**	121	594	102	344	223	937
**non-parent**	71	130	92	131	163	261
**total**	192	724	194	475	386	1199

Table S15. Number of individuals and urine samples by grandparent status included in the grandparent model.

	**female**		**male**		**total**	
	n individuals	*n* samples	*n* individuals	*n* samples	*n* individuals	*n* samples
**grandparent**	46	305	35	118	81	419
**non-grandparent**	145	418	160	354	305	775
**total**	191	723	195	472	386	1195

Table S16. Number of individuals and urine samples by maternal grandfather status included in the maternal grandparent model.

	**female**		**male**		**total**	
	n individuals	*n* samples	*n* individuals	*n* samples	*n* individuals	*n* samples
**grandparent**	40	288	28	96	68	384
**non-grandparent**	151	435	167	376	318	811
**total**	191	723	195	472	386	1195

Table S17. Number of individuals and urine samples by local paternal grandfather status included in the paternal grandparent model.

	**female**		**male**		**total**	
	n individuals	*n* samples	*n* individuals	*n* samples	*n* individuals	*n* samples
**grandparent**	30	211	25	87	55	298
**non-grandparent**	161	512	170	385	331	897
**total**	191	723	195	472	386	1195

Table S18. Number of individuals and urine samples by sex and self-rated health status included in the seflf-rated health model.

	**female**		**male**		**total**	
	n individuals	*n* samples	*n* individuals	*n* samples	*n* individuals	*n* samples
**bad**	14	116	2	5	16	121
**normal**	49	183	38	104	87	287
**good**	14	39	10	27	24	66
**total**	77	338	50	136	127	474
